# Ambient microdroplet annealing of nanoparticles†

**DOI:** 10.1039/d1sc00112d

**Published:** 2021-03-24

**Authors:** Angshuman Ray Chowdhuri, B. K. Spoorthi, Biswajit Mondal, Paulami Bose, Sandeep Bose, Thalappil Pradeep

**Affiliations:** DST Unit of Nanoscience (DST UNS), Thematic Unit of Excellence (TUE), Department of Chemistry, Indian Institute of Technology Madras Chennai 600 036 India pradeep@iitm.ac.in

## Abstract

Conversion of polydisperse nanoparticles to their monodisperse analogues and formation of organized superstructures using them involve post synthetic modifications, and the process is generally slow. We show that ambient electrospray of preformed polydisperse nanoparticles makes them monodisperse and the product nanoparticles self-assemble spontaneously to form organized films, all within seconds. This phenomenon has been demonstrated with thiol-protected polydisperse silver nanoparticles of 15 ± 10 nm diameter. Uniform silver nanoparticles of 4.0 ± 0.5 nm diameter were formed after microdroplet spray, and this occurred without added chemicals, templates, and temperature, and within the time needed for electrospray, which was of the order of seconds. Well organized nanoparticle assemblies were obtained from such uniform particles. A home-made and simple nanoelectrospray set-up produced charged microdroplets for the generation of such nanostructures, forming cm^2^ areas of uniform nanoparticles. A free-standing thin film of monodisperse silver nanoparticles was also made on a liquid surface by controlling the electrospray conditions. This unique method may be extended for the creation of advanced materials of many kinds.

## Abstract 2

Polydisperse silver nanoparticles were converted to a highly ordered assembly of nanoparticles by microdroplet-induced chemistry, under ambient conditions, within seconds.

## Graphical abstract

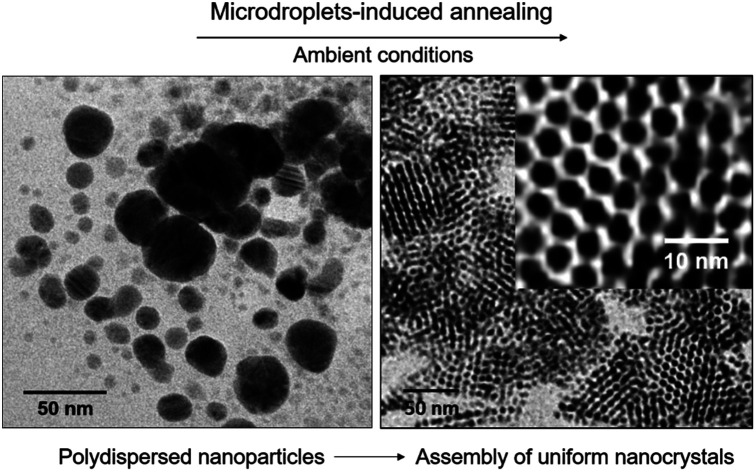

## Introduction

Chemical processes in microdroplets are a rapidly evolving subject. Examples include synthesis of molecules^1,2^ and pharmaceutical products^3^ as well as conformational changes in proteins.^4,5^ Such synthesis can also produce nanoparticles (NPs) without reducing agents starting from metal ions in solutions.^6–8^ This synthetic method can be tuned with the application of an electrical potential and can lead to assemblies of nanomaterials, and a viable method for forming 1D structures with potential applications was demonstrated.^7^ Materials science with charged microdroplets can produce nanoholes on 2D materials^9^ and metallic thin films on liquid surfaces.^10^

The science of nanomaterials, especially noble metal nanomaterials, has expanded into almost every area of materials science.^11–14^ The properties of these materials are heavily dependent on their size, shape, and distribution. These aspects are especially important for their electronic structure and consequently applications involving chemistry, physics, and biology.^14^ As a result, several methods have been developed to control the size dispersity of such materials. Digestive ripening is one of the most commonly used methods in this regard for noble metal NPs.^15–17^ The method typically involves high-temperature annealing involving refluxing of NP suspensions for extended periods to achieve monodispersity.^15,18,19^ However, extremely precise conditions for a long time, of the order of days, are required to obtain uniform particles. Therefore, it is important to develop a facile and fast method for making monodisperse nanostructures.

Ultrafast acceleration of chemical reactions and synthesis of NPs starting from metal precursors in droplets suggest that new chemical bonds of diverse kinds can be formed and broken under such synthetic conditions.^1,2,6–8^ This prompted us to explore the possibility of spontaneous dissociation and reassembly of preformed NPs in microdroplets. To our surprise, such droplet-induced dissociation and reassembly of polydisperse silver NPs protected with thiols resulted in highly monodisperse NPs in the microsecond time scale. The deposition of such monodisperse particles produced a film of uniform NPs, and this process is millions of times faster than digestive ripening. Exploring the science through a series of control experiments showed that metal thiolates are transient precursor species formed in this process. We have also optimized conditions under which such a process is feasible to make cm^2^ area films of uniform particles. As this method is similar to high-temperature annealing, leading to monodispersity, we term it microdroplet annealing.

## Results and discussion

### Observation of microdroplet annealing

In the present experiment, we have utilized a home-built nanoelectrospray source to deliver charged microdroplets containing polydisperse 2-phenylethanethiol (PET)-protected silver NPs (Ag@PET NPs) in dichloromethane (DCM) onto a transmission electron microscopy (TEM) grid placed on an indium tin oxide (ITO)-coated collector plate. The collector was grounded through a picoammeter to monitor the deposition current, and a potential in the range of 4.5–5.0 kV was applied to the solution held within a glass spray tip through a platinum (Pt) wire electrode. The spray plume was ejected from the nanoelectrospray tip, which can be visualized with a laser torch. Further details are available in the experimental section and ESI.† Various other substrates could be used in place of the TEM grid (see below).

**Fig. 1:**
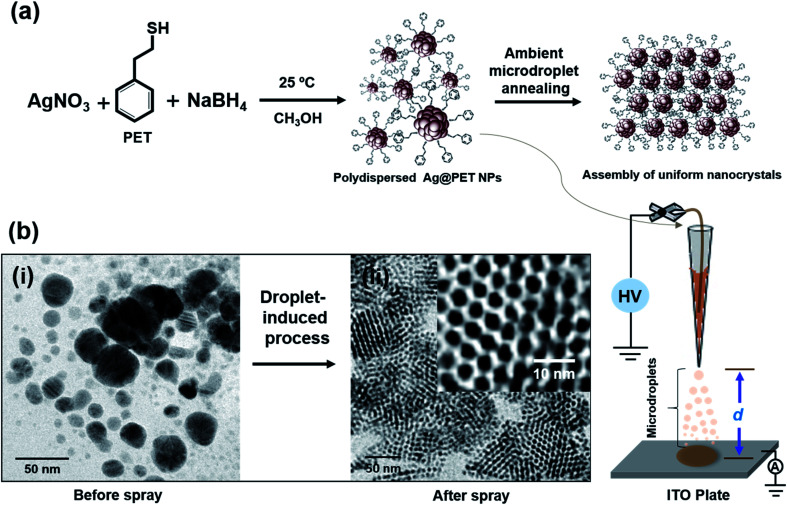
Schematic representation of (a) the synthetic route of polydisperse Ag@PET NPs and the experimental process leading to the conversion of polydisperse NPs to monodisperse analogues by ambient microdroplet annealing. The experimental set-up for ambient microdroplet annealing is presented schematically in the bottom panel. (b) TEM images of Ag@PET NPs before and after electrospray, respectively (inset of b(ii) shows a corresponding expanded view of the TEM image). Electrospray tip-to-collector distance (*d*) was kept at 1.5 cm.

In the present work, an organized assembly of uniform NPs was formed on the TEM grid, starting from the corresponding polydisperse NPs. The as-synthesized Ag@PET NPs have a broad size distribution, as shown schematically in Fig. 1a. The particle size distribution covers a broad range, from 2–25 nm, as determined by TEM (Fig. 1b(i)). This distribution is typical of such NPs. Further details of the characterization of the starting material are presented in the ESI (Fig. S1†). A *d*-spacing of 0.23 nm is due to Ag(111) and it suggested the growth of pure metallic NPs.^20^ The characteristic Fourier-transform infrared spectroscopy (FTIR) features of the as-synthesized Ag@PET NPs confirm the attachment of PET on the AgNPs (Fig. S1c, ESI†). The as-synthesized Ag@PET NPs exhibit prominent surface plasmon resonance at 451 nm (Fig. S1d, ESI†). Details of synthesis and characterization are also presented in the experimental section and ESI.†

Ag@PET NPs in DCM were transferred to a home-built borosilicate spray capillary of 50 μm inner diameter (ID) for microdroplet-induced reaction. A schematic representation of the home-built electrospray set-up is shown in Fig. 1. In the course of electrospray deposition of Ag@PET NPs, a dark circular spot of 0.5 cm diameter due to the impinging plume appeared on the substrate kept at a distance *d* from the spray tip, shown in the bottom panel of Fig. 1. The drastic change observed in the course of the reaction is shown in Fig. 1b. Positively charged microdroplets convert polydisperse Ag@PET NPs to an ordered 2D superlattice structure of uniform NPs.^21–28^ A superlattice in the current context refers to a localized periodic assembly of uniform NPs which possess inherent periodicity of the element. Such structures are possible only with uniform particles, and the Ag@PET NPs formed have a particle size distribution of 4.00 ± 0.50 nm, which is evident from the expanded portion of the TEM image (inset of Fig. 1b(ii)). From separate experiments it is known that droplet formation and deposition occurs in hundreds of microseconds to milliseconds.^3,29–31^ Surprisingly, microdroplet chemistry *via* ambient electrospray converts irregular particles into a superstructure of uniform particles within a fraction of a second.^6–10^

### Optimizing microdroplet annealing

**Fig. 2:**
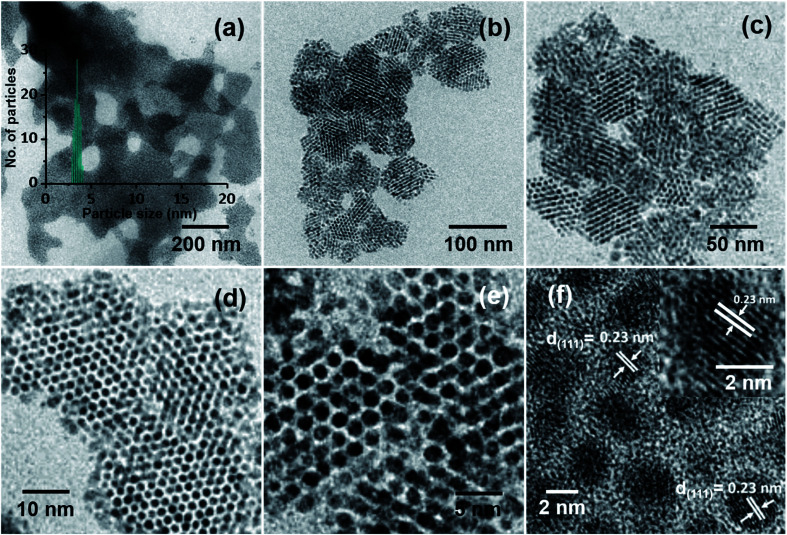
HRTEM images (at different magnifications) of Ag@PET NPs after electrospray under optimized conditions. (a) Large-area TEM image, showing a 2D sheet-like morphology, the particle size distribution obtained is shown in the inset. Expanded images: (b and c) assembly leading to superlattices, (d and e) TEM images showing uniform inter-particle distance, and (f) the (111) lattice plane of Ag@PET NPs; the HRTEM image of a particle is expanded in the inset.

In the process of optimization, a series of trials were made to achieve ordered NP assemblies. Finally, ordered structures of Ag@PET NPs were obtained at an applied voltage of 5 kV, *d* of 1.5 cm and flow rate of 1.0 mL h^−1^, and the data are shown in Fig. 2. A large-area view of monodisperse Ag@PET NPs is shown in Fig. 2a, which form a nearly continuous uniform 2D structure. The corresponding magnified images are shown in Fig. 2b–f, which portray the orientation of the superlattice domains. The inter-particle gap in the superlattice structure was ∼1.2 nm (Fig. 2e), slightly less than the length of two PET ligands (the distance between S and Ph–C4 in the PET ligand is ∼0.7 nm (ref. 32)), suggesting ligand interactions driving the assembly. Ligands usually form interdigitated structures in these types of assemblies.^33^ The Ag(111) lattice spacing of 0.23 nm (Fig. 2f) and the TEM-energy dispersive spectroscopy (TEM-EDS) data (Fig. S2†) confirm the formation of crystalline AgNPs. The FTIR spectrum (Fig. S2b†) shows that PET protection remains intact after such ambient solution-state conversion. The reduced width of the optical absorption spectrum confirms the narrowness of particle sizes after electrospray (Fig. S2a†). This abrupt change in particle distribution post-electrospray suggests a digestive ripening-type of phenomenon through microdroplet chemistry. Inside the microdroplets, polydisperse Ag@PET NPs of size between 2 and 25 nm get reorganized and become uniform particles of 4.00 ± 0.50 nm size (inset of Fig. 2a). Images at different magnifications (Fig. 2b–d) suggest that there is scope for the formation of a 3D superlattice assembly due to overlayer deposition. Negatively charged microdroplets did not produce such superlattice assemblies.

Events within microdroplets can be modified by altering *d*, under a particular electric field. Monodispersity was achieved at *d* = 1–2.5 cm at a spray voltage of 5 kV (Fig. S3b–d, ESI†). At shorter and longer *d*, polydispersity was seen, as shown in Fig. S3a and e–h, ESI†. This observation suggests that at *d* < 1 cm there was not enough time for the reorganization of the NPs in the microdroplets. At *d* > 2.5 cm, droplets were not producing NP assembly owing to the destabilization of microdroplets, due to evaporation of solvents. It could be demonstrated that when *d* was 1.5 cm, Ag@PET NPs achieved the perfect condition for self-assembly leading to superlattices. An optimum structure was obtained at 1.5 cm, and hence this *d* was maintained for other experiments. After fixing *d*, the electrospray voltage was optimized between 0.5 and 7.0 kV. In this process, the electric field and deposition current of microdroplets were adjusted accordingly. The monodisperse assembly of Ag@PET NPs was obtained at 5.0 kV (Fig. S4e, ESI†). The rate of flow of Ag@PET NPs from the spray emitter was also an essential parameter. The size of microdroplets depended on the flow rate. For the given condition, microdroplets produced ordered structures at a flow rate of 1.0 mL h^−1^, as shown in Fig. S5b, ESI.†

Solvent plays a vital role in the generation of a suitable environment within the microdroplets. The generation of the spray-plume and materials formed in microdroplets are highly dependent on the polarity of the solvent. The dielectric constant (*ε*), pH, and pressure of the droplet environment are important electrospray parameters.^31,34^ In this regard, solvents with different *ε* were studied under the above-optimized conditions to check the effect of solvents on the formation of such structures within microdroplets. Monodispersity was achieved for a few solvents having a particular range of *ε* under optimized conditions in the case of PET-protected silver NPs, as shown in Fig. S6c–g.† Organized assembly was observed when such a process was continued with DCM having an *ε* of 8.93. Other solutions, with solvents such as carbon tetrachloride, *ε* = 2.24, diethyl ether, *ε* = 4.33, chloroform, *ε* = 4.81, tetrahydrofuran, *ε* = 7.82, pyridine, *ε* = 12.40, acetone, *ε* = 20.70, dimethylformamide, *ε* = 37.50, and acetonitrile, *ε* = 38.25 were unable to produce such monodisperse assembly. This study was continued with four different capillaries with IDs in the range 30–60 μm under the optimized conditions. An ID of 50 μm was most appropriate under these conditions.

### Extension to other ligands

It is clear that an unusual phenomenon in microdroplets was observed with PET protected AgNPs under a particular condition. In order to check the possibility of such transformations with other protecting ligands, trials were made with different ligands, especially thiols with different denticities and chain lengths under the optimized conditions achieved for Ag@PET NPs. Different polydisperse AgNPs were synthesized with 2,4-dimethylbenzenethiol (DMBT) and water-soluble sodium citrate (Cit), and their TEM images are shown in Fig. S7a and c, ESI.† Previously optimized conditions did not produce monodispersity in these cases (Fig. S7b and d, ESI†). Polydisperse dithiol-protected AgNPs were synthesized with 1,4-benzenedithiol (BDT) and 1,6-hexanedithiol (HDT), and spray experiments were performed subsequently. In the case of Ag@BDT NPs, monodispersity without such assembly was seen (Fig. S8a and b, ESI†). However, spray of Ag@HDT NPs was inefficient even to produce a significant change in the particle size, as presented in Fig. S8c and d.† These experiments were extended to comparatively long-chain thiols like octadecanethiol (ODT)- and dodecanethiol (DDT)-protected AgNPs. A broad size distribution was observed after the spray of ODT and DDT-protected AgNPs (Ag@ODT NPs & Ag@DDT NPs), which may be due to the difficulty in reorganization of long carbon chain thiols within microdroplets (Fig. S9, ESI†). Optimization of spray conditions is necessary to get monodisperse assemblies for these NP systems. However, ambient microdroplet annealing produced monodisperse assembly of Ag@DMBT NPs at 8.5 kV and *d* = 1.5 cm, as shown in Fig. S10.† The method was also successful in creating uniform NPs of ethanethiol (ET)-protected Ag NPs from polydisperse NPs at an applied voltage of 4.0 kV and *d* = 1.0 cm (Fig. S11†). Therefore, it is clear that this process is general and is applicable for different NP systems under specific electrospray conditions. Optimization of spray conditions is essential for a particular NP system. The concentration of polydisperse Ag@PET NPs taken for the spray experiments is also important. Fig. S12 of the ESI† shows the TEM images of Ag@PET NPs after spraying at different concentrations. These experiments demonstrate that uniformity is dependent on the starting concentration of the polydisperse Ag NPs. In summary, monodispersity needs optimization of a range of parameters.

### Nebulization spray

**Fig. 3:**
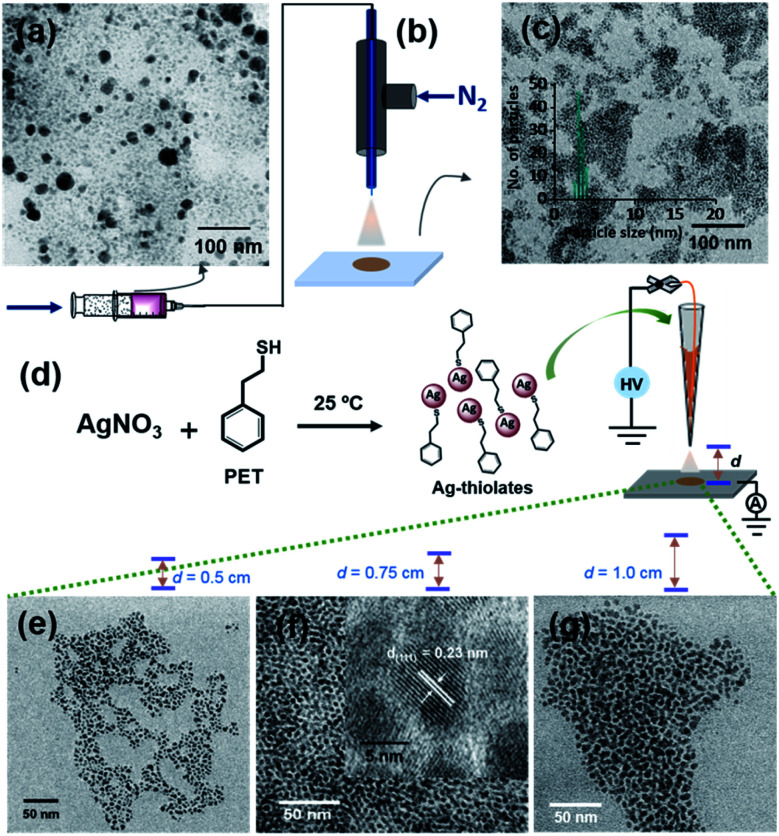
Generation of monodisperse Ag@PET NPs from polydisperse Ag@PET NPs using microdroplets. (a) TEM of the as-synthesized Ag@PET NPs, (b) schematic representation of the spray of polydisperse Ag@PET NPs using dry N_2_ gas without an electric field; the ID of the silica microcapillary was 50 μm, the same as the spray tip used in the electrospray. (c) TEM image of monodisperse Ag@PET NPs after spray (size distribution is in the inset), (d) schematic of the formation of soluble Ag–PET thiolates and their electrospray at three different *d* values, (e–g) TEM images of Ag@PET NPs obtained at a *d* of 0.50 cm, 0.75 cm, and 1.00 cm, respectively. Nearly ordered assemblies are formed from thiolates at *d* = 0.75. An expanded view of the same image is shown in the inset of f, showing the Ag(111) lattice.

The aerosol spray can also be produced by nebulization.^31,34^ This process does not require an applied electric potential. Ag@PET NPs (Fig. 3a) were sprayed using dry N_2_ gas with four different pressures, using a microcapillary of 50 μm diameter. A schematic of this spray set-up is presented in Fig. 3b. Microdroplets produced in the spray can create uniform particles with an average particle size of 4 nm, as shown in Fig. 3c. Thus, monodispersity can be achieved by voltage-free spray as well. However, the spray does not produce uniform ordered superstructures, which may be due to the high-pressure of N_2_ gas from the emitter. This may require additional optimization of the pressure needed. Fig. S13† shows the TEM images of Ag@PET NPs after spray at different N_2_ nebulization pressures. For all these cases, a nearly similar particle size distribution was observed.

### Thiolate intermediates

A probable mechanism for the formation of such organized structures was hypothesized with inputs from control experiments. We know that Ag^+^ and thiols in solution form thiolates during the formation of NPs^35,36^ and clusters.^37,38^ Silver thiolates are formed when clusters decompose.^39,40^ Therefore, we propose that silver thiolates are formed in droplets as intermediates during the formation of uniform NPs. To prove this, we conducted a spray experiment by taking Ag–PET thiolates as precursors (see the experimental section for their synthesis), instead of polydisperse NPs. Thiolates were sprayed at three different *d* values (Fig. 3e–g). All the experiments produced silver NPs, but the organized structures with silver lattice planes were seen at *d* = 0.75 cm (Fig. 3f). This suggests that microdroplet spray of solvated polydisperse particles resulted in highly ordered assemblies, most likely through a transient thiolate species, as presented in Fig. S14.†

### Large-area films

**Fig. 4:**
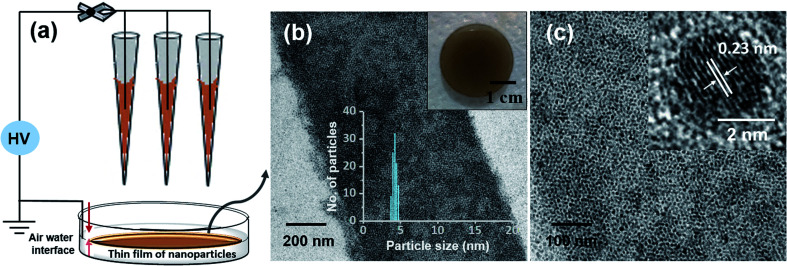
(a) Schematic illustration of the electrospray deposition of Ag@PET NPs on a water surface. The formation of a thin film of Ag@PET NPs on a liquid surface is also shown, (b) a large-area TEM image of monolayer Ag@PET NPs from the deposited film (inset: photograph of the free standing film deposited on water after spray and its particle size distribution), (c) HRTEM image of NPs which confirms the ordered structure and formation of metallic Ag particles. The well-defined Ag(111) lattice is shown in the inset of c.

This ambient ion-based methodology has been introduced for the creation of free-standing metallic monodisperse nanoparticles–nanosheets (NPs–NSs). In an earlier study, we electrodeposited metal salts on a liquid surface and got self-organized films of the particles under the influence of electrohydrodynamic flow.^10^ A similar approach was used here. The set-up, schematically represented in Fig. 4a is the same as that shown in Fig. 1, except that multiple nozzles and a liquid substrate were used. The multi-nozzle electrospray enlarges the magnitude of the NPs–NSs on the liquid substrate. In this objective, a series of trials were performed using different solvents. It was observed that among the solvents studied, the ordered NP structure was formed on the water surface. These uniform NPs-NSs, composed of superlattice structures of silver NPs, can be used for diverse applications. The large-area TEM image in Fig. 4b confirms the compactness of the assembly. The particle size distribution was in the range of 4.5 ± 0.5 nm. The formation of a brown colored thin film of NPs–NSs was observed and is shown in the inset of Fig. 4b. Fig. 4c shows the corresponding magnified image. The lattice spacing of these NPs–NSs matches with the (111) plane of AgNPs, proving the metallic nature of AgNPs. Similarly, polydisperse NPs were deposited on an ITO surface under optimized spray conditions and were redispersed in DCM. A uniform film of monodisperse NPs was observed using a TEM, as shown in Fig. S16 (ESI†).

**Fig. 5:**
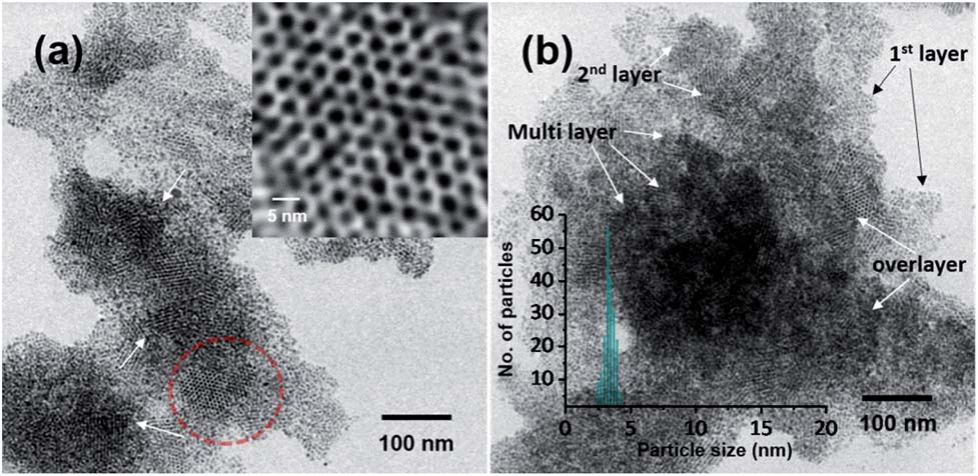
Formation of 3D ordered monodisperse assemblies of AgNPs by electrospray for an extended period. (a) TEM image of Ag@PET NPs after 10 min of electrospray, which shows the deposition of layers of NPs (an expanded view of the TEM image is shown in the inset). Genesis of a second layer of superlattices is shown by the arrows. (b) TEM image of Ag@PET NPs after 15 min of electrospray (inset: particle size distribution). More layers are seen. Arrows indicate the formation of multiple layers to create 3D superlattices.

Polydisperse silver NPs can also form 3D assemblies on a metal substrate as shown in Fig. 5a. If we look closely at the structures, a layer-upon-layer assembly of nanoparticles is observed, which is indicated by arrows in Fig. 5b. During spray for an extended period, the incoming microdroplets with the transient metal-thiolates are continuously reduced to NPs and get deposited on preexisting nanoparticle layers.^7^ The 3D crystal structures formed this way could generate exceptional properties.^41,42^ Fig. S17 in the ESI† shows the growth of a multilayer assembly with respect to time. The developed superlattice structures can be used as an ink for electrospray-based printing of a range of crystalline materials. This ambient approach can also print bulk composites and porous architectures. The simplicity of this ambient process is expected to create interest for the development of such 3D structures directly from polydisperse NPs.

A series of control experiments were performed to optimize the ordered structure by varying different parameters, especially, spray distance (*d*), voltage and flow rate. The experiment was repeated under the optimized conditions. It was confirmed that an electric field and a deposition current of ∼35 nA were also vital for the creation of such nanostructures. This methodology may provide an opportunity to produce large scale monodisperse silver NP assemblies *via* such ambient microdroplet annealing. Moreover, this ambient technique could certainly be used as a facile synthetic approach for the development of unique nanomaterials of other materials with emerging properties for a broad range of applications. The possibility of assembly over large areas along with uniform synthesis offers new possibilities.

## Conclusions

In summary, we developed a fast method of making monodisperse silver NPs by electrospraying highly polydisperse NPs synthesized in solution, under ambient conditions. Microdroplets from the spray formed a uniform assembly of nanostructures composed of ordered AgNPs upon deposition on a substrate. Control experiments proved that the electric field, the tip-to-collector distance, and the flow rate are the key factors for the oriented growth of such superlattices. This process does not require any sophisticated instrumentation, and it transforms polydisperse NPs to superlattices composed of monodisperse NPs within seconds. This process may be considered green as no solvents or additional processing is involved, unlike solution state post-processing methods. Microdroplet spray on a water surface makes a thin film of monodisperse metallic sheets of uniform NPs assisted by electrohydrodynamic flow. The method described in this work may be utilized for the development of multimetallic superlattice structures or high entropy alloys by efficient control of the composition of metals, leading to new properties.

## Experimental

### Materials and chemicals used

Silver nitrate (AgNO_3_) was purchased from RANKEM. Sodium borohydride (NaBH_4_), 2-phenylethanethiol (PET), 1,4-benzenedithiol (BDT), 2,4-dimethylbenzenethiol (DMBT), sodium citrate (Cit), ethanethiol (ET), 1-octadecanethiol (ODT), and dodecanethiol (DDT) were purchased from Sigma Aldrich Chemicals. Pure ethanol, methanol, dichloromethane, pyridine, carbon tetrachloride, acetone, diethyl ether, chloroform, acetonitrile, and *N*,*N*-dimethylformamide were sourced from Merck India and used as solvents for the electrospray deposition experiments.

### Synthesis of PET protected silver NPs (Ag@PET NPs)

Polydisperse Ag@PET NPs were synthesized by our previously described method with slight modifications.^33^ Briefly, PET (0.58 μL) was added to pure methanol (30 mL) at 25 °C. Subsequently, AgNO_3_ (50 mg) in Millipore water (0.5 mL) was added to the above solution. The mixture was gently stirred for 15 min to form silver thiolates. After that, NaBH_4_ (25 mg) was dissolved in 8 mL ice-cold water and was subsequently added slowly into the flask and stirred for another 12 h to allow the complete growth of Ag@PET NPs. The as-synthesized Ag@PET NPs were washed with methanol and extracted in different solvents for further experiments.

### Synthesis of other ligand protected silver NPs

Different ligand protected AgNPs were synthesized for comparative experiments. BDT, DMBT, ET, HDT, Cit, ODT, and DDT were used separately during the synthesis of AgNPs in individual trials. For every case, the experimental procedure was similar, except for the addition of a particular thiol in methanol at the beginning of the synthesis. After synthesis, the products were washed with methanol and extracted in DCM for electrospray experiments.

### Electrospray deposition experiments

A home-made electrospray set-up was used for the generation of microdroplets (Fig. 1a).^7^ A micropipette puller (P-97, Sutter Instruments, U.S.A.) was used for pulling a borosilicate glass capillary of 1.5 mm outer diameter (OD) and 0.86 mm ID. It was cut into two pieces having tips of 50 μm ID and 150 μm OD. Each tip of the capillary was checked using an optical microscope to ensure the size and quality of the cut. Polydisperse Ag@PET NPs (100 μg mL^−1^) in DCM were filled in the nanoelectrospray tips using a microinjector pipette tip, and Pt wire (0.5 mm diameter) was inserted into the solution, making an electrode for high voltage connection. A multi-nozzle spray set-up was used for scaling up the method. A syringe pump controlled the flow rate of the microdroplets to achieve the best structures. The syringe needle was connected to a high voltage power source through a copper clip. The experiments were carried out in different solvents and solvent mixtures having different *ε* and at varying potentials. Among the solvents used, spray with DCM showed the best result. More control experiments were carried out with silver thiolates in DCM. ITO-coated glass slides were procured from Aldrich. Millipore water was used in all the experiments.

### Microdroplet generation through nebulizer gas

For nebulization spray, a home-built set-up was used as presented in the schematic (Fig. 3b). A Hamilton syringe was connected to a silica capillary (50 μm ID) through a union connector. Microdroplets were generated by spraying solvents through a fused silica capillary of 50 μm ID with 10, 20, 30, and 40 pounds per square inch (psi) N_2_ gas for nebulization.

### Characterization of the nanostructures

TEM and HRTEM were performed at an accelerating voltage of 200 kV on a JEOL 3010, 300 kV instrument equipped with a UHR polepiece. A Gatan 794 multiscan CCD camera was used for image acquisition. Energy-dispersive X-ray spectroscopy (EDS) spectra were collected on an Oxford Semistem system housed on the TEM. The formation of NPs during microdroplet deposition was examined directly using 300-mesh carbon-coated copper grids (spi Supplies, 3530C-MB) under different experimental conditions. Silver NPs were deposited directly on the TEM grids under different experimental conditions. Particle size distributions were obtained from TEM images using ImageJ.

## Author contributions

The idea was suggested by TP. ARC and SB performed most of the experiments. The manuscript was written through contributions of all authors. All the authors approved the final version of the manuscript.

## Conflicts of interest

There are no conflicts to declare.

## Supplementary Material

SC-012-D1SC00112D-s001
