# Supplementary material for: Ambient microdroplet annealing of nanoparticles
Source: Chem Sci. 2021 Mar 24;12(18):6370–7. doi: 10.1039/d1sc00112d (PMC8115297; doi:10.1039/d1sc00112d)
Supplement: SC-012-D1SC00112D-s001 [file SC-012-D1SC00112D-s001.pdf]

Electronic Supplementary Material (ESI) for Chemical Science.
This journal is © The Royal Society of Chemistry 2021

Electronic Supplementary Information

Ambient Microdroplet Annealing of Nanoparticles

*Angshuman Ray Chowdhuri, B. K. Spoorthi, Biswajit Mondal, Paulami Bose, Sandeep Bose, Thalappil Pradeep**

DST Unit of Nanoscience (DST UNS) and Thematic Unit of Excellence (TUE), Department of Chemistry, Indian Institute of Technology Madras, Chennai - 600036, India

*Email: pradeep@iitm.ac.in

Table of contents

Name	Description	Page No.
Fig. S1	Characterization of polydispersed Ag@PET NPs	S2
Fig. S2	UV-Vis, FTIR spectra, and EDS of Ag@PET NPs before and after electrospray	S3
Fig. S3	Optimization of *d* for the formation of organized assemblies	S4
Fig. S4	Optimization of the applied voltage for the formation of organized assemblies	S5
Fig. S5	Flow rate of microdroplets optimization for the formation of organized assemblies	S6
Fig. S6	TEM images of NPs after spray in different solvents	S7
Fig. S7	TEM images of as-synthesized Ag@DMBT NPs, Ag@Cit NPs and their corresponding product NPs after electrospray	S8
Fig. S8	TEM images of as-synthesized Ag@BDT NPs, Ag@HDT NPs and their corresponding product NPs after electrospray	S9
Fig. S9	TEM images of as-synthesized Ag@ODT NPs, Ag@DDT NPs and their corresponding product NPs after electrospray	S10
Fig. S10	TEM image of organized assemblies of Ag@DMBT NPs at optimized spray conditions for Ag@DMBT NPs	S11
Fig. S11	TEM image of organized assemblies of Ag@ET NPs at optimized spray conditions for Ag@ET NPs	S12
Fig. S12	Concentration optimization of Ag@PET NPs for the formation of organized assemblies	S13
Fig. S13	Spray of polydispersed Ag@PET NPs by using dry N_2 gas	S14
Fig. S14	Schematic of probable mechanism for the formation of organized assemblies	S15
Fig. S15	Measurement of charge of microdroplets during electrospray	S16
Fig. S16	Optical and TEM images of NPs film deposited on ITO	S17
Fig. S17	Development of 3D assemblies of silver nanoparticles by electrospray	S18

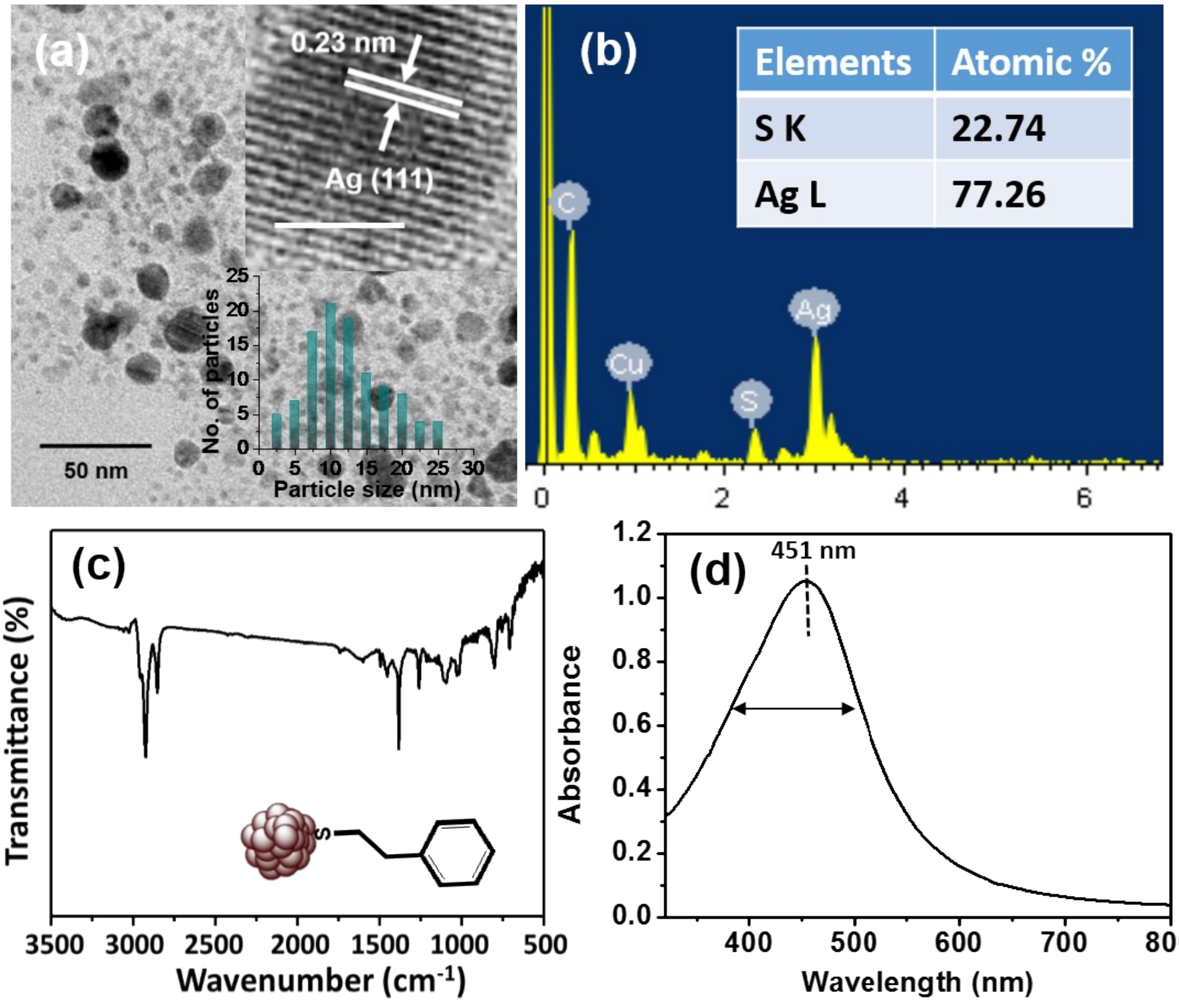

Fig. S1 Characterization of as-synthesized polydispersed Ag@PET NPs. (a) TEM image of the as synthesized polydispersed Ag@PET NPs (insets: HRTEM of a particle showing Ag(111), scale bar is 2 nm, and the observed particle size distribution), (b) TEM-EDS spectrum, (c) FTIR spectrum exhibits the attachment of PET with the AgNPs. The absence of S-H stretch at 2560 cm^{-1} is due to the binding of the thiol on the silver surface. Result indicated the loss of thiolate proton and RS-Ag bond formation in the NPs. (d) UV-Vis spectrum of Ag@PET NPs shows the plasmonic feature at 451 nm.

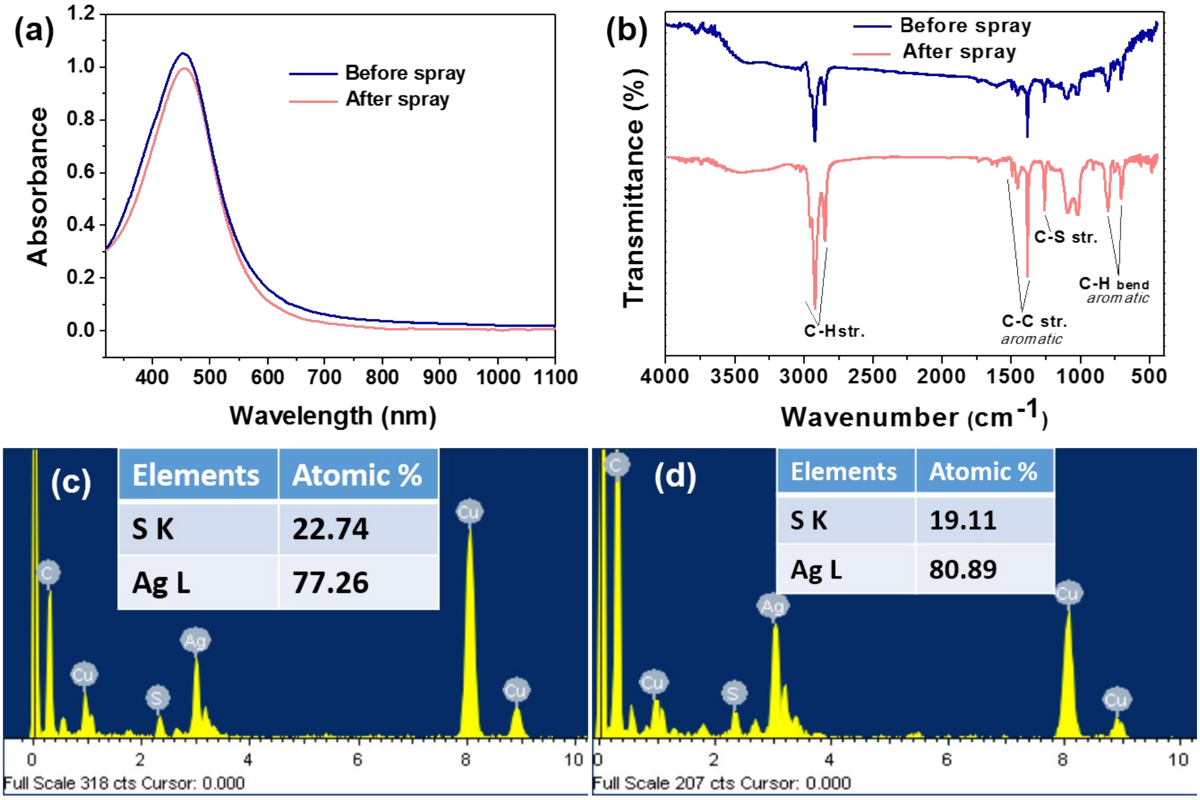

Fig. S2 (a) UV-Vis, (b) FTIR spectra of Ag@PET NPs before and after electrospray. After spray, the NPs were collected from ITO and dissolved in DCM for UV-Vis study. The FITR spectrum after electrospray suggests that PET remains intact on the silver assemblies after the spray. The characteristic peaks in the EDS of Ag@PET NPs (c) before and (d) after electrospray.

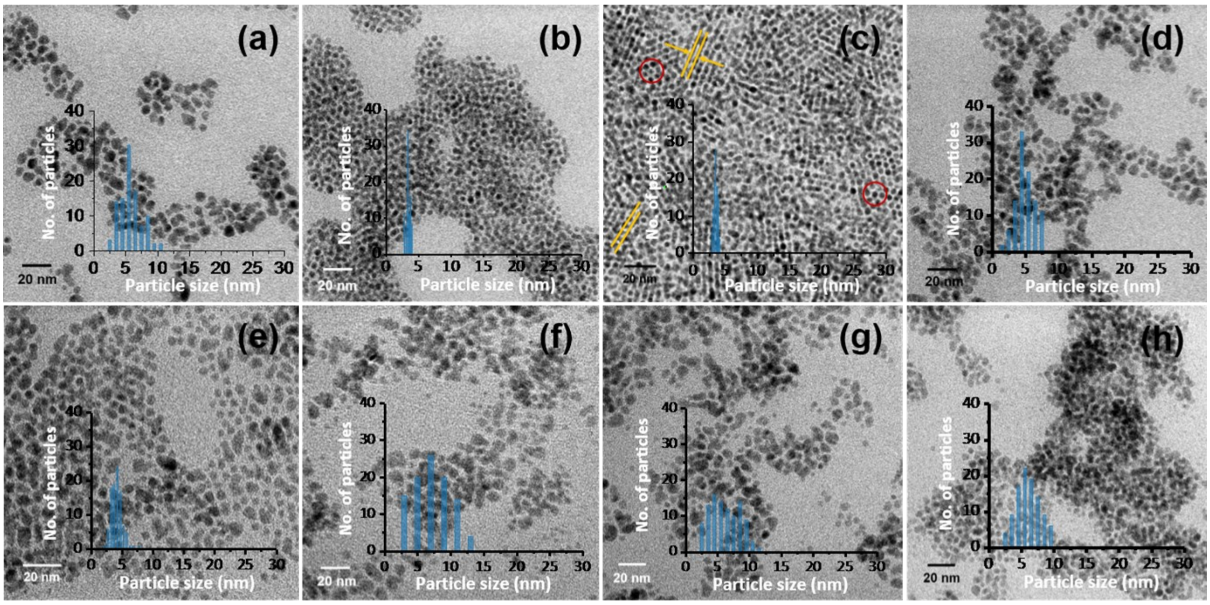

Fig. S3 Optimization of *d* for the formation of monodispersed assemblies of Ag@PET NPs. TEM images of the NPs while *d* was kept at (a) 0.5, (b) 1.0, (c) 1.5, (d) 2.0, (e) 2.5, (f) 3.0, (g) 3.5, and (h) 4.0 cm, during electrospray.

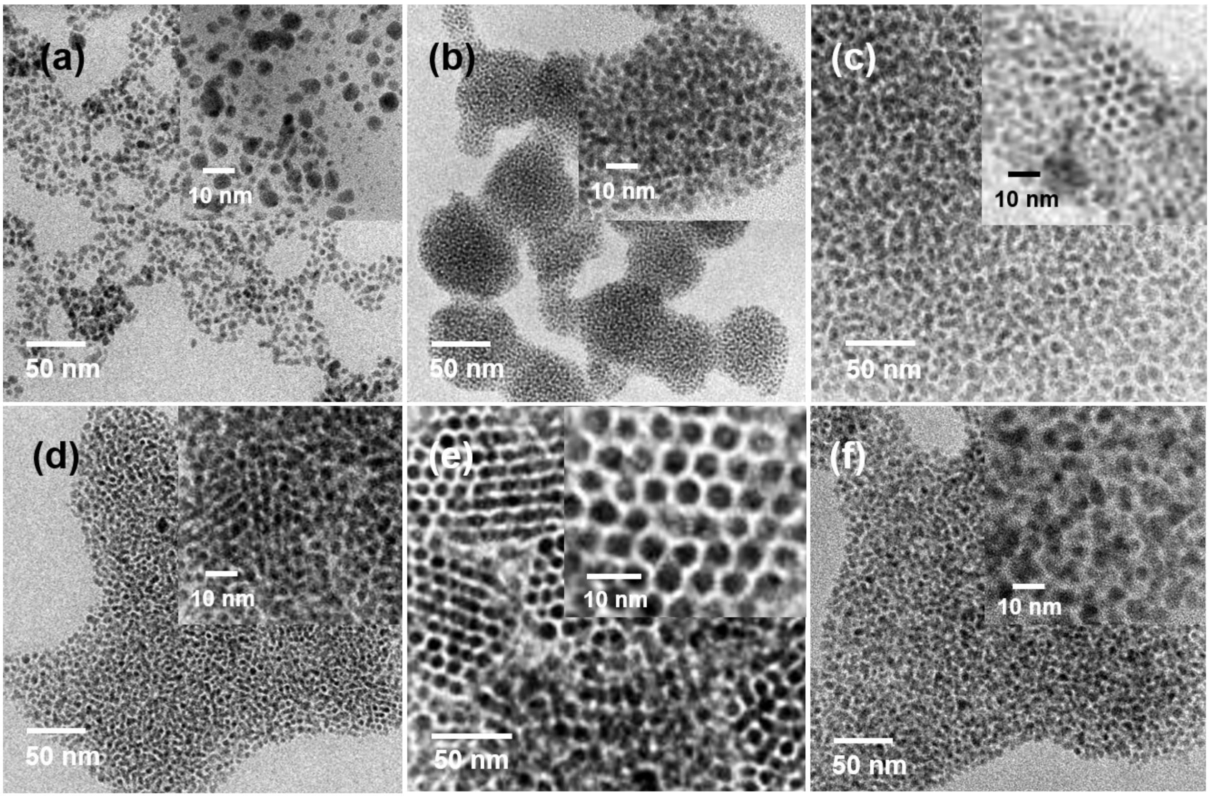

Fig. S4 Optimization of the applied voltage for the process. TEM images of NPs at applied voltages of (a) 0.5, (b) 1.0, (c) 2.0, (d) 3.0, (e) 5.0, and (f) 7.0 kV.

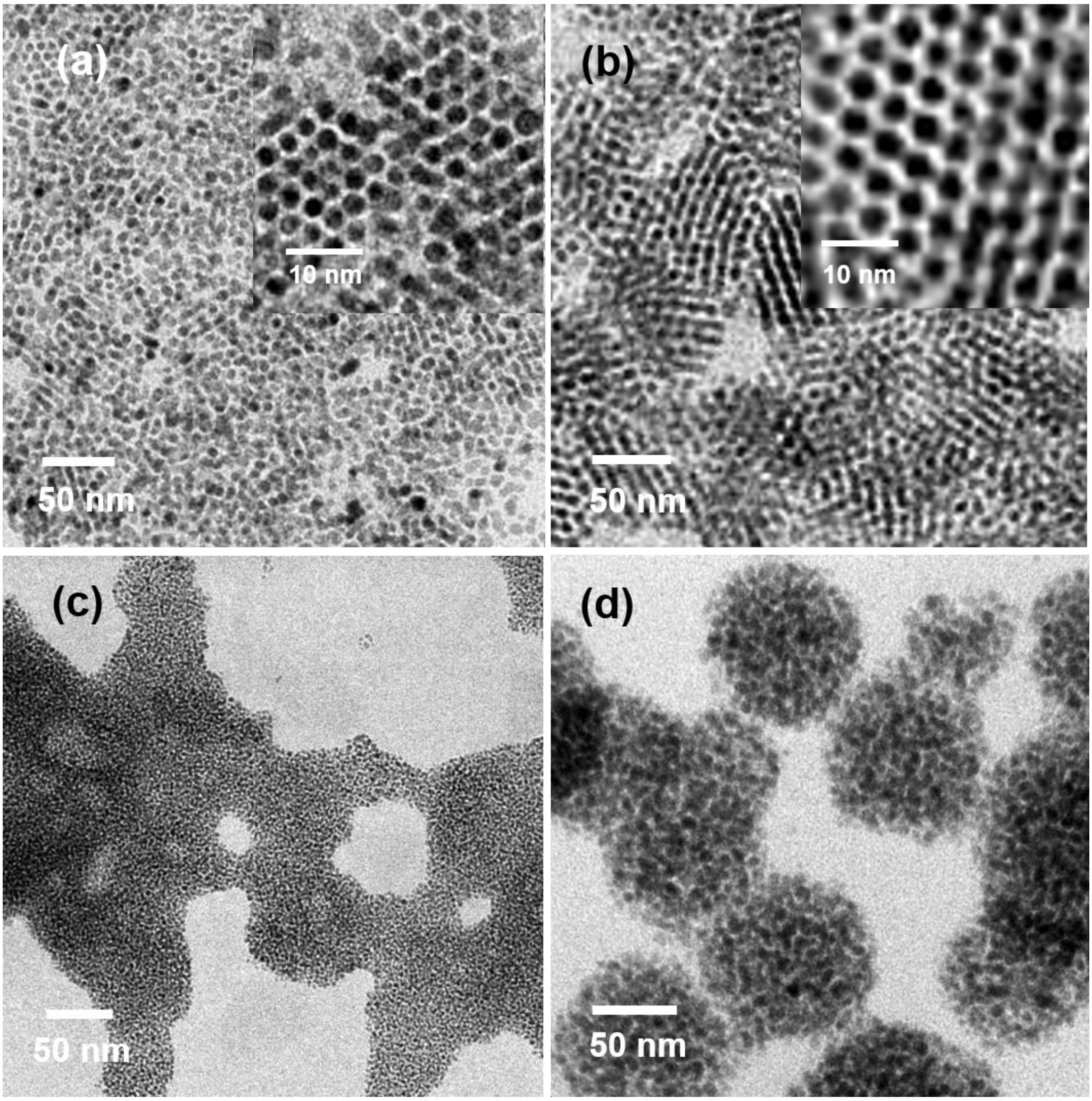

Fig. S5 TEM images of Ag@PET NPs after electrospray with the flow rates of (a) 0.50, (b) 1.00 (optimized conditions, presented in Figure 1b), (c) 1.25, and (d) 1.50 mL/h.

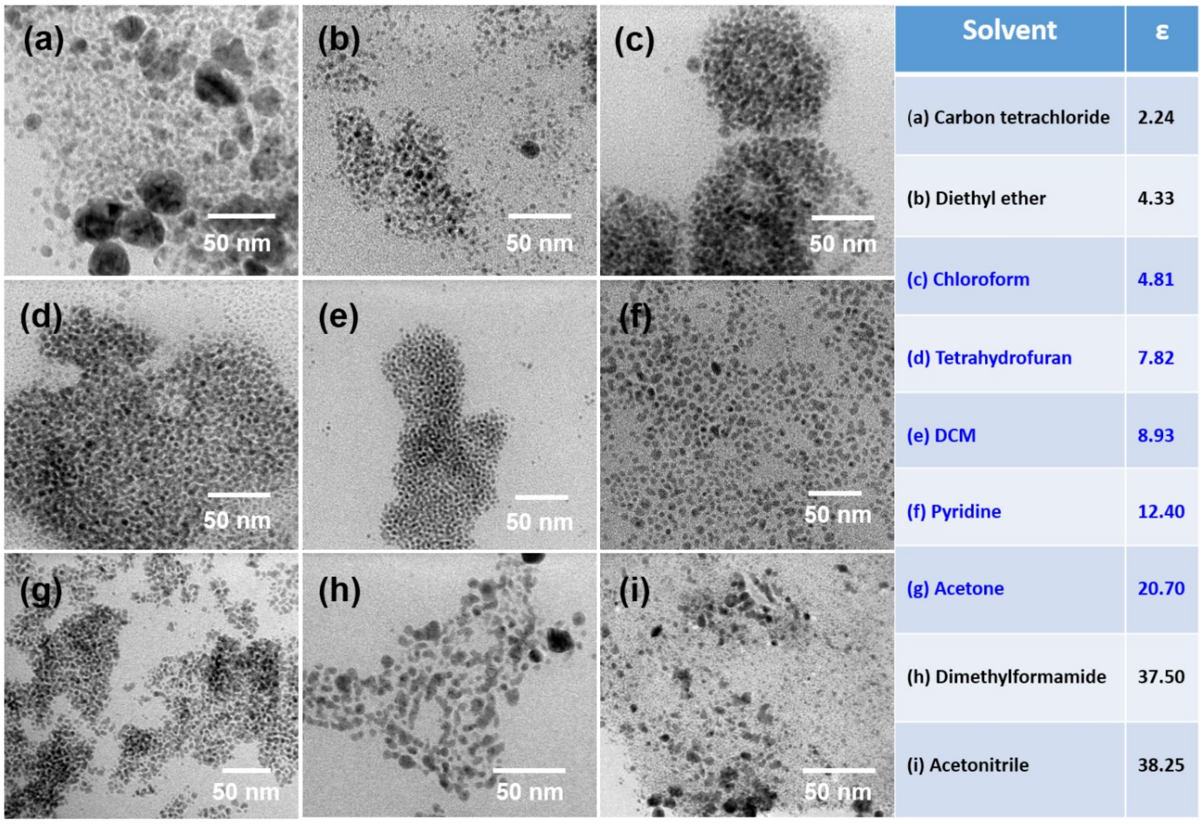

Solvent	ε
(a) Carbon tetrachloride	2.24
(b) Diethyl ether	4.33
(c) Chloroform	4.81
(d) Tetrahydrofuran	7.82
(e) DCM	8.93
(f) Pyridine	12.40
(g) Acetone	20.70
(h) Dimethylformamide	37.50
(i) Acetonitrile	38.25

Fig. S6 Electrospray of Ag@PET NPs in different solvents having lower to higher dielectric constant (ε) is studied. The environment (pH, pressure, charge) inside the microdroplet is changing with the physical and chemical properties (polarity, surface tension, viscosity) of the solvents used for electrospray. TEM images of Ag@PET NPs after electrospray in (a) carbon tetrachloride, (b) diethyl ether, (c) chloroform, (d) tetrahydrofuran, (e) DCM, (f) pyridine, (g) acetone, (h) dimethylformamide, and (i) acetonitrile. The ε values of solvents are presented in the table. Monodispersed NPs were achieved between ε = 4.81–20.70, as shown in Figure c-g.

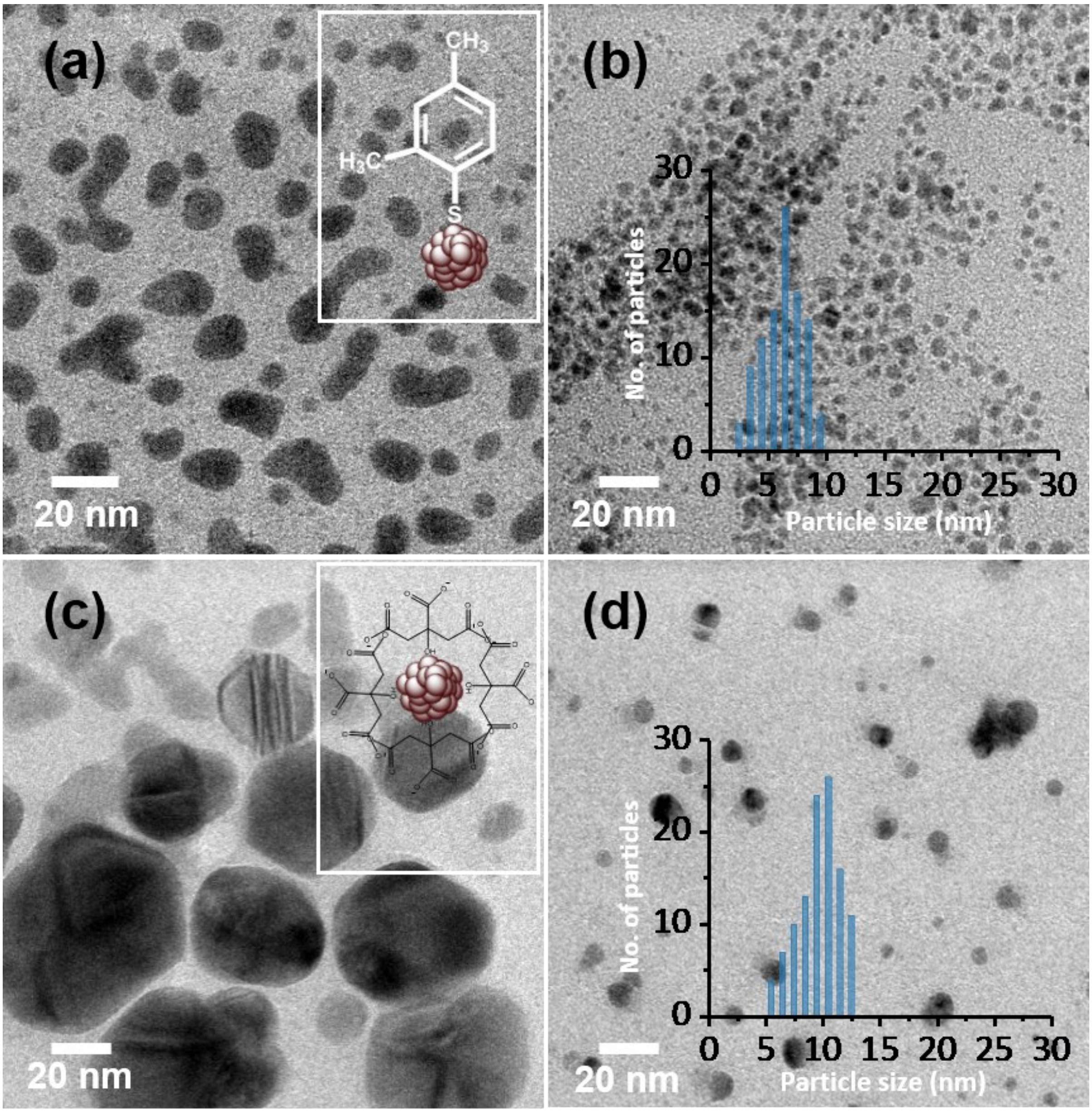

Fig. S7 TEM images of as-synthesized (a) Ag@DMBT NPs and (c) Ag@Cit NPs. Images of the corresponding product NPs after electrospray are in (b) and (d), respectively, at optimized condition used for Ag@PET NPs. The particle size distribution after spray is presented in the inset. Monodispersity was not achieved.

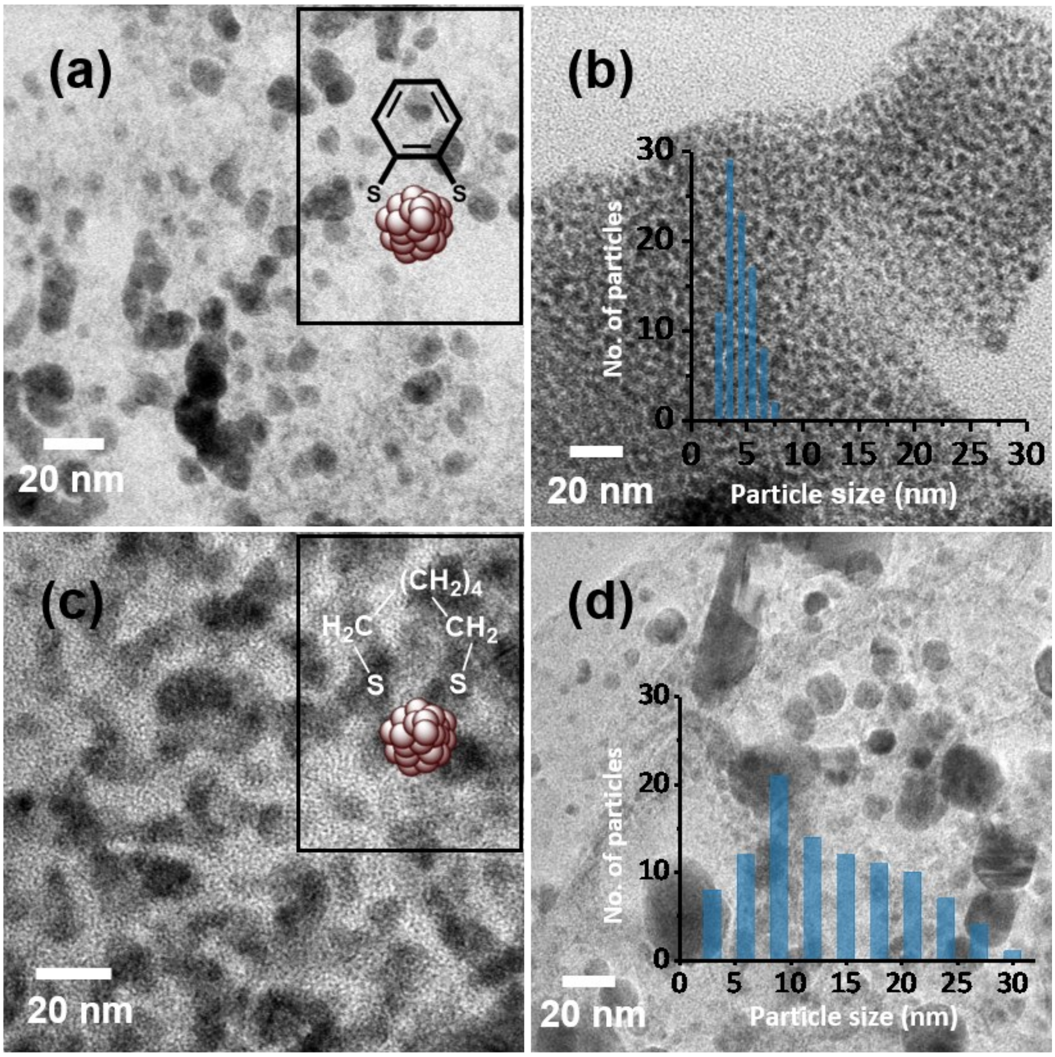

Fig. S8 TEM images of as-synthesized (a) Ag@BDT NPs and (c) Ag@HDT NPs. Images of the corresponding product NPs after electrospray are in (b) and (d), respectively, at optimized condition used for Ag@PET NPs. Particle size distribution in inset b suggests the particles tend to achieve monodispersity without forming superlattices.

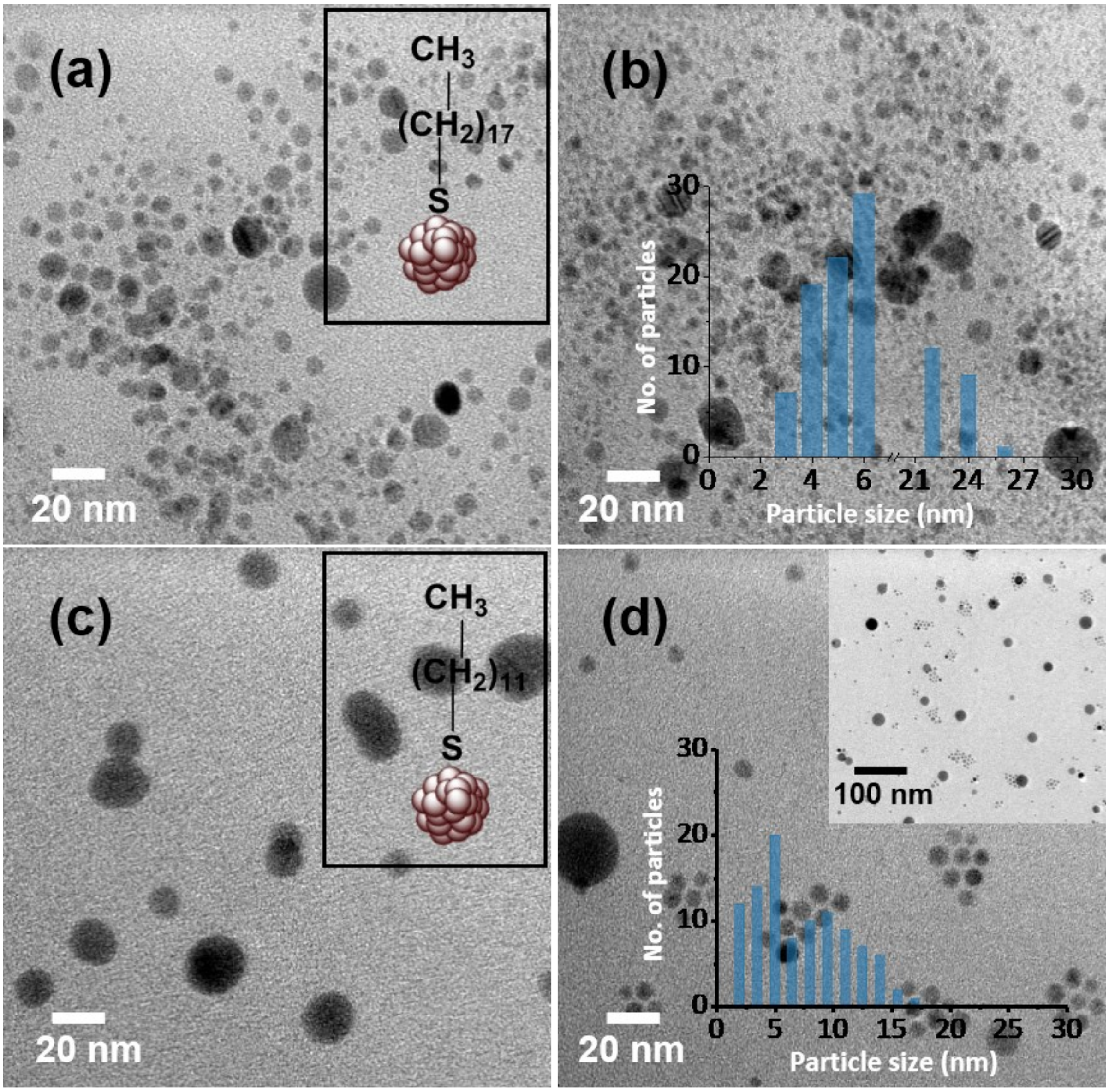

Fig. S9 TEM images of as-synthesized (a) Ag@ODT NPs and (c) Ag@DDT NPs. Images of the corresponding product NPs after electrospray are in (b) and (d), respectively, at optimized condition used for Ag@PET NPs.

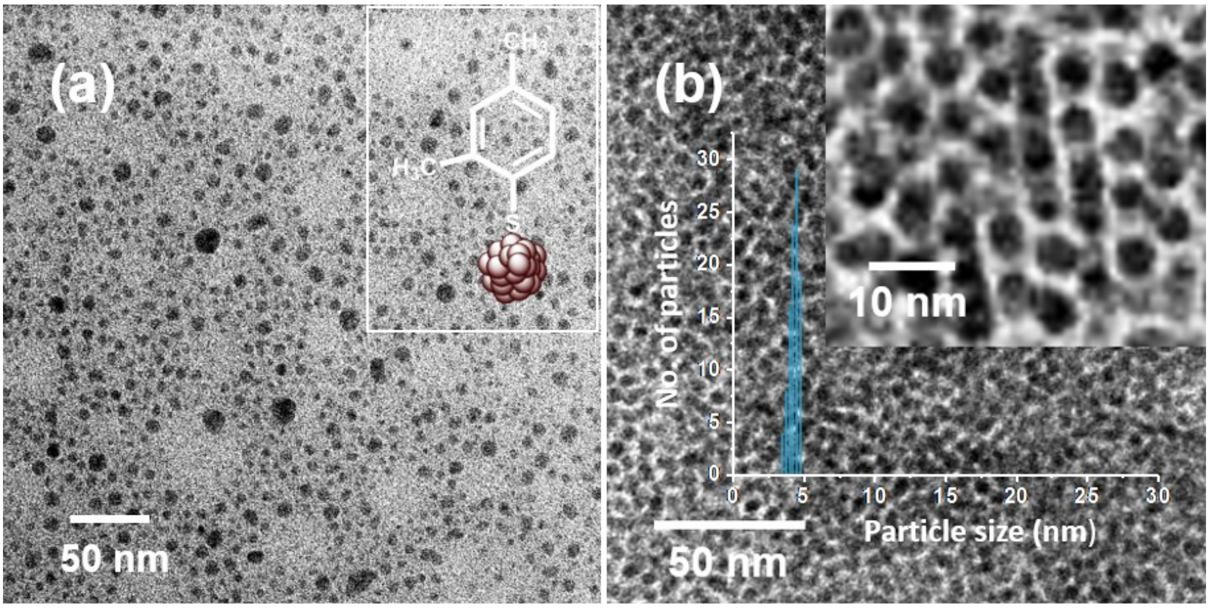

Fig. S10 TEM images of as-synthesized (a) Ag@DMBT NPs. (b) Corresponding monodispersed NPs after electrospray at applied voltage of 8.5 kV, d = 1.5 cm. Expanded view of the TEM image and particle size distribution are presentated in the inset. Uniform assembly of NPs of 4.25 ± 0.50 nm was observed after the spray. The ambient microdroplet annealing process was capable of producing such ordered assembly of uniform Ag@DMBT NPs at optimized spray conditions.

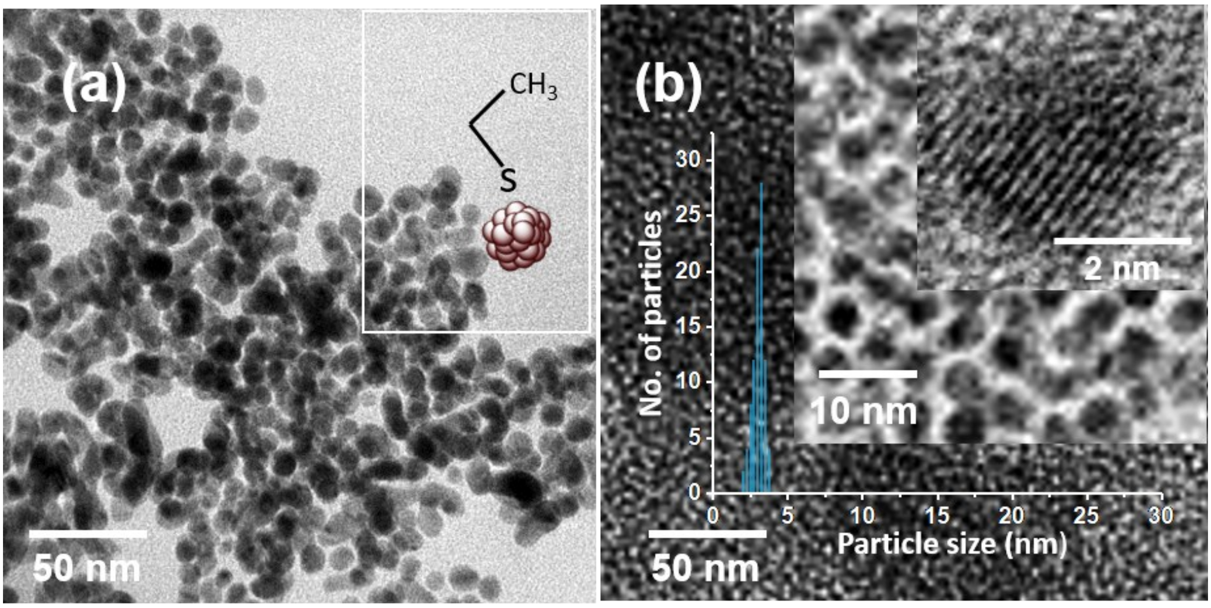

Fig. S11 TEM images of as-synthesized (a) Ag@ET NPs. (b) Corresponding monodispersed NPs after electrospray at applied voltage of 4 kV, d = 1.0 cm. Expanded view of the TEM images and particle size distribution are presentated in the inset. Monodispersed NPs of 3.0 ± 1.0 nm were obtained after the spray. Uniform assembly of NPs was achieved by the ambient microdroplet annealing.

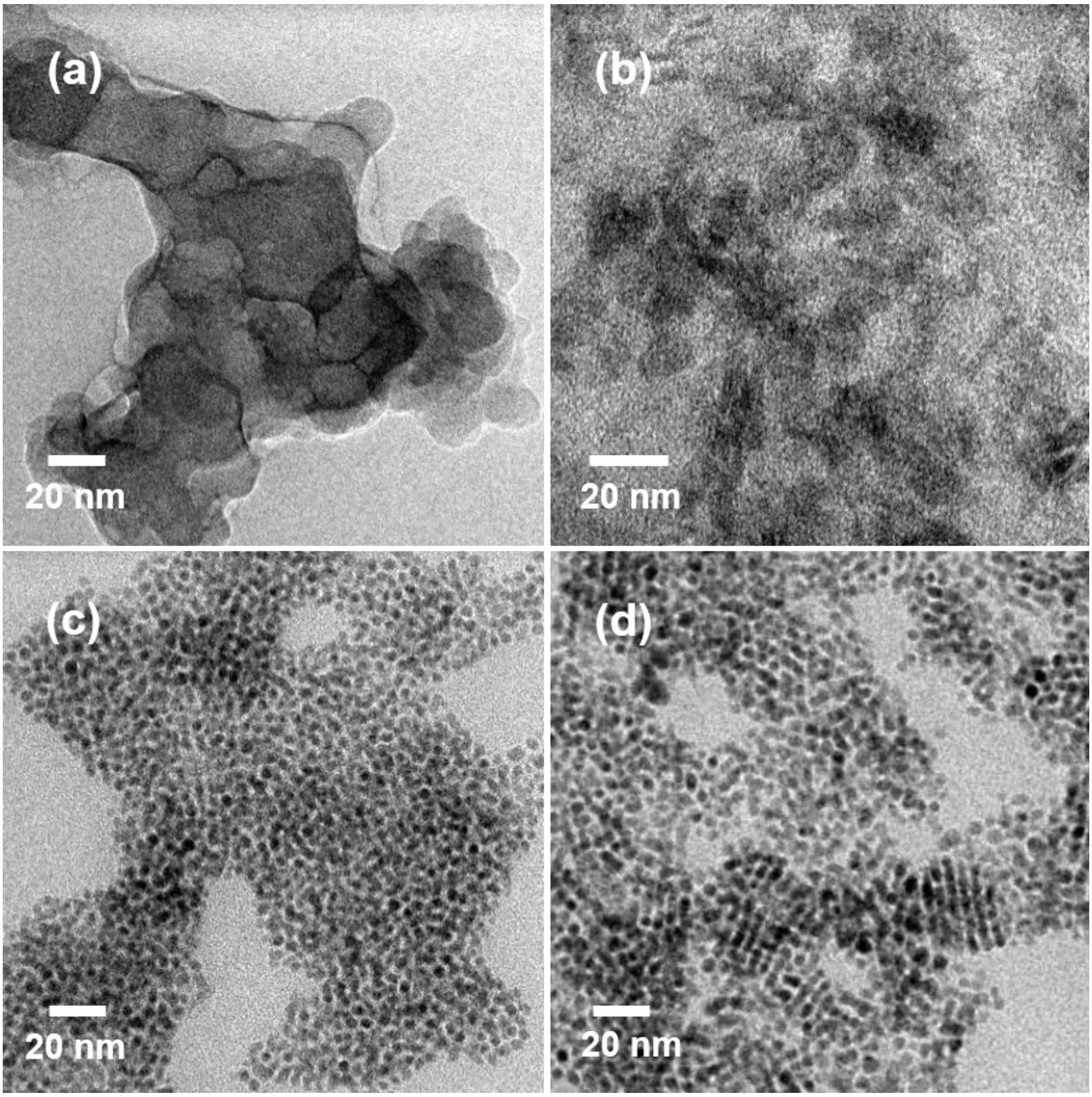

Fig. S12 Variation of concentration of as-synthesized Ag@PET NPs for the creation of organized assemblies. TEM images of the species obtained from (a) 25, (b) 50, (c) 75, and (d) 125 μg/mL concentration of polydispersed Ag@PET NPs. About 100 μg/mL of NPs produced best result as presented in Figure 2. Result revealed that this process is dependent on the concentration of Ag@PET NPs.

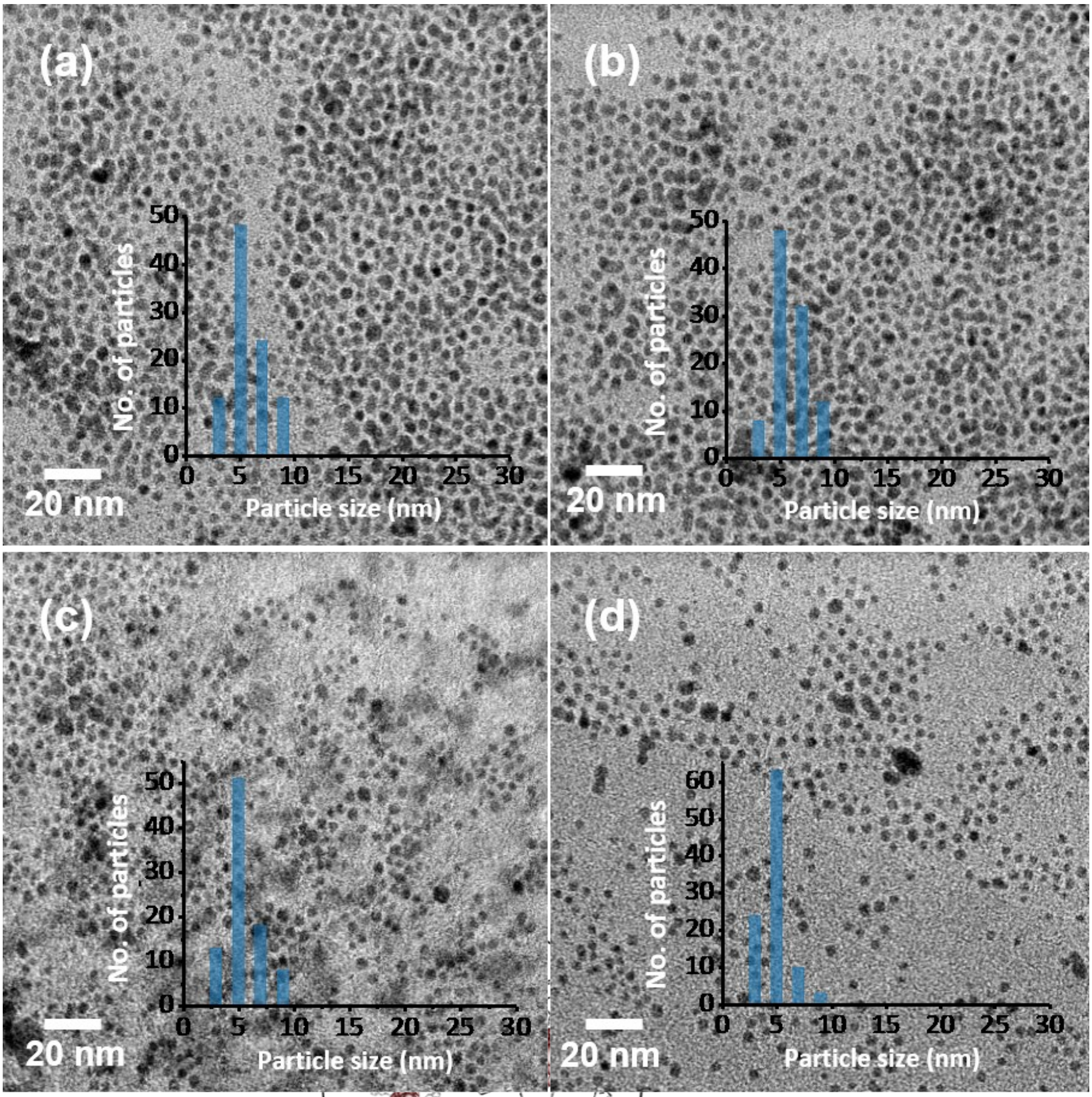

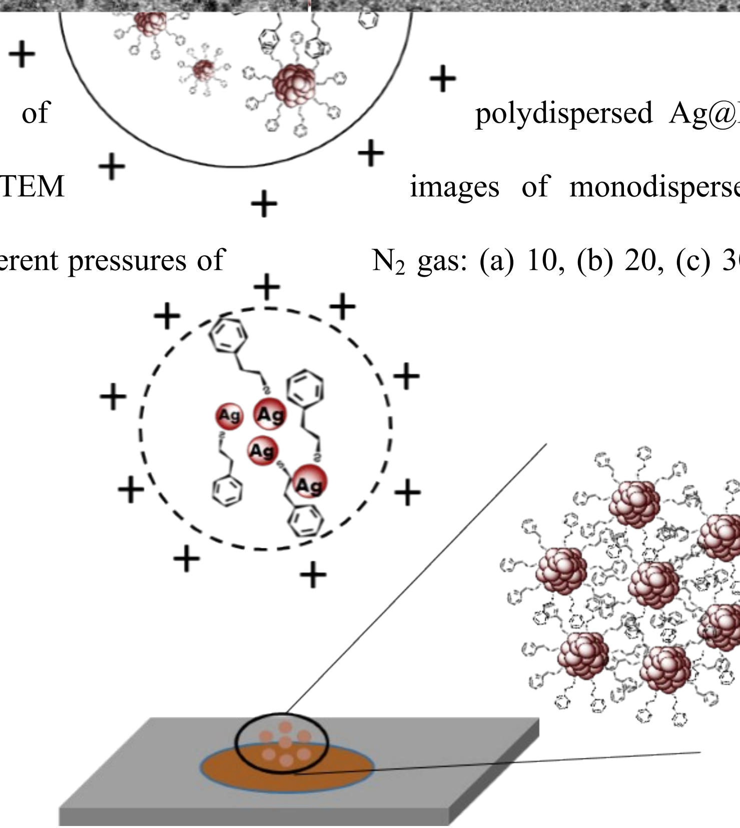

Fig. S13 Spray of polydispersed Ag@PET NPs by using dry N_2 gas. TEM images of monodispersed Ag@PET NPs after spray at different pressures of N_2 gas: (a) 10, (b) 20, (c) 30, and (d) 40 psi.

Fig. S14 Schematic representation of a probable mechanism for the formation of monodispersed Ag@PET NPs and their well-defined assemblies via droplets. Our nebulization experiments show that charge on the droplet was not essential.

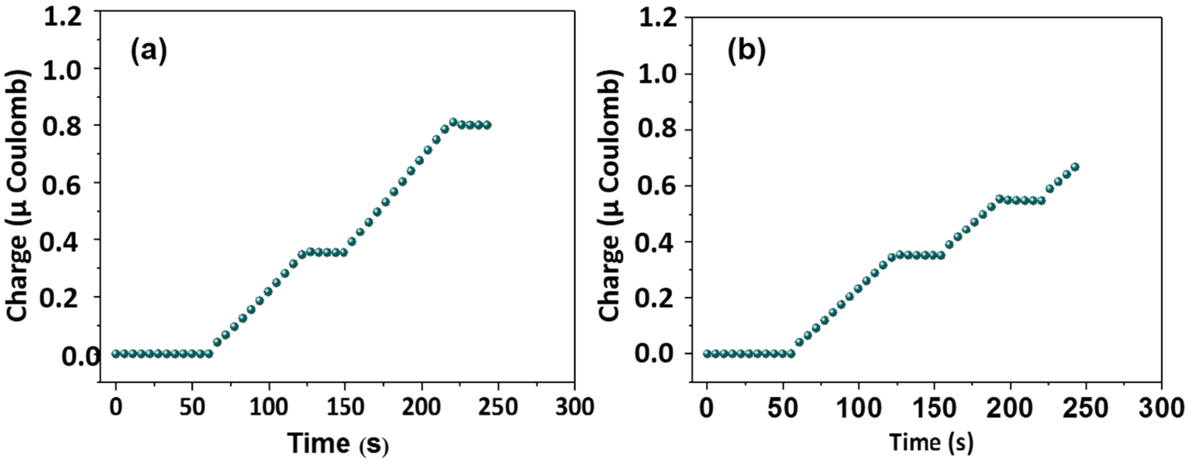

Fig. S15 Measurement of charge of microdroplets loaded with Ag@PET NPs during electrospray deposition with respect to time at *d* of (a) 1.0 cm and (b) 1.5 cm. During the course of electrospray, the charge of the ITO plate (substrate) was measured continuously with respect to time using an electrometer. The charge was increasing upon deposition of positively charged microdroplets on the ITO plate. This experiment was carried out to test the charge of the droplet. It was important to mention that there was no charge accumulation on the substrate during spray. Similar experiments were carried out for *d* of (a) 1.0 cm and (b) 1.5 cm. When the charge was constant, there was no spray, as spray happened in short intervals.

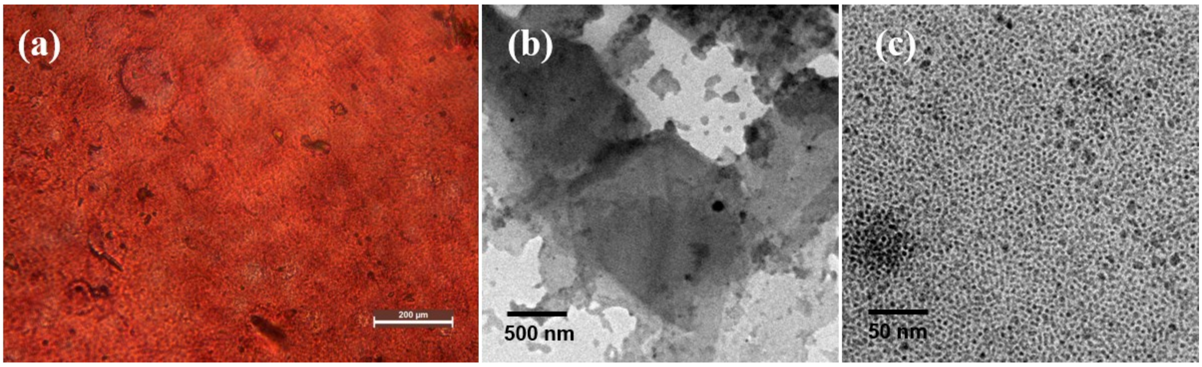

Fig. S16 (a) Optical microscope image of deposited film on ITO surface. Deposited NPs on ITO surface is redispersed in DCM and placed on carbon-coated copper grids. (b) TEM image of NPs film deposited on ITO surface and (c) corresponding high magnification image.

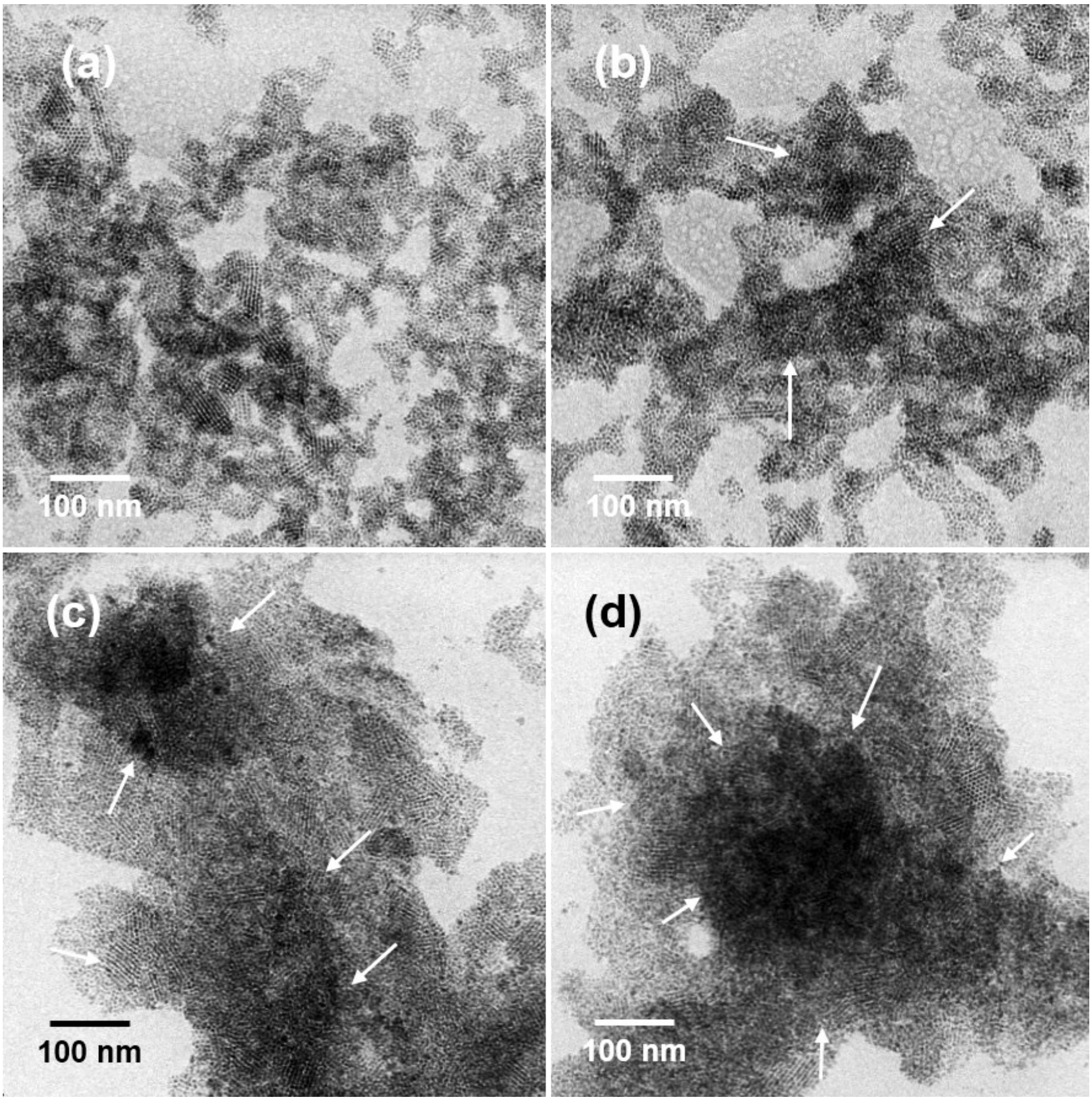

Fig. S17 Formation of 3D assemblies during different times of spray; (a) 4, (b) 6, (c) 8, and (d) 15 min. More layers were generated with respect to time. White arrows indicate the formation of overlayer structures.
